# Implementation of mobile EEG for resting-state and visual evoked potentials in young children in rural Ethiopia

**DOI:** 10.3389/fnhum.2025.1552410

**Published:** 2025-06-10

**Authors:** Theresa I. Chin, Winko W. An, Kalkidan Yibeltal, Firehiwot Workneh, Stephen Pihl, Sarah K. G. Jensen, Gellila Asmamaw, Nebiyou Fasil, Atsede Teklehaimanot, Krysten North, Sonya V. Troller-Renfree, Charles A. Nelson, Yemane Berhane, Anne CC Lee

**Affiliations:** ^1^Division of Biology and Medicine, Warren Alpert Medical School of Brown University, Providence, RI, United States; ^2^Harvard Medical School, Boston, MA, United States; ^3^Department of Pediatrics, Brigham and Women’s Hospital, Boston, MA, United States; ^4^Boston Children’s Hospital, Boston, MA, United States; ^5^Department of Reproductive Health and Population, Addis Continental Institute of Public Health, Addis Ababa, Ethiopia; ^6^Department of Epidemiology and Biostatistics, Addis Continental Institute of Public Health, Addis Ababa, Ethiopia; ^7^Department of Global Health and Health Policy, Addis Continental Institute of Public Health, Addis Ababa, Ethiopia; ^8^College of Health Science, Tikur Anbessa Specialized Hospital, Addis Ababa University, Addis Ababa, Ethiopia; ^9^Department of Human Development, Teachers College, Columbia University, New York, NY, United States

**Keywords:** electroencephalography (EEG), neurodevelopment, low- and middle-income countries (LMIC), nutrition, visual evoked potential (VEP)

## Abstract

Children living in low- and middle-income countries (LMIC) are at disproportionately higher risk of neurodevelopmental delays due to exposure to adverse biological and environmental hazards. In infancy, global developmental assessments, such as the Bayley Scales, are insensitive, do not strongly correlate with later cognitive outcomes, and require adaptation for different populations and cultural contexts. Electroencephalography (EEG) objectively measures electrical brain activity and may provide early neural markers predictive of long-term cognitive outcomes. The visual evoked potential (VEP) interrogates the efficiency of visual cortical processing and reflects neural processing speed. Mobile EEG enables the assessment of neural processing in settings where such technologies were historically inaccessible. This paper describes the experiences and lessons learned from implementing mobile EEG and VEP in rural Amhara, Ethiopia as part of the Longitudinal Infant Growth and Development (LIDG) study (NCT06296238). We describe adaptations and strategies to address and optimize data capture (e.g., dry electrode tips to improve scalp contact, tailored protocols, and adequate equipment specifications), environmental challenges (e.g., space constraints, lack of water supply, power outage) and cultural factors (e.g., hair type) unique to the study setting and population. Our formative research underscored the importance of creating awareness among community members (e.g., mothers, fathers, and religious leaders) and local clinicians to improve community engagement and buy-in. Culturally sensitive child behavior management techniques were also critical to ensure EEG completion and high data quality. With community sensitization, we had high consent rates for EEG/VEP (>90%). We completed EEG recordings within an average ± standard deviation of 20 ± 11 minutes. After data processing, approximately 90% and 70% of participants met predefined data quality thresholds for resting EEG and VEP, respectively. Implementing mobile EEG/VEP was feasible and acceptable in rural Ethiopia, with a relatively high proportion of recordings meeting quality standards.

## Introduction

The “first 1,000 days,” spanning from conception to two years of age, is characterized by a period of rapid brain development. During this period, the brain is highly susceptible to the cumulative and enduring effects of risk factors, including poverty, nutrient deficiencies, inflammation, environmental pollution, and limited access to healthcare (Walker et al., 2007). The higher rate of these risk factors in low- and middle-income countries (LMIC) contributes to a staggering burden of 250 million children who do not reach their developmental potential by age five, globally, a majority of whom reside in sub-Saharan Africa (Black et al., 2017). At the same time, this period also represents a critical window during which the brain is most amenable to health and psychosocial interventions to optimize long-term developmental outcomes. The evaluation of intervention efficacy relies on well-established standards of typical neurodevelopmental trajectories. However, the paucity of objective and cross-culturally valid measures to define these trajectories in LMIC may hinder accurate assessments of intervention effects and early detection of possible developmental delays, particularly in infancy. Global developmental assessments are widely utilized in LMIC. However, in infancy, tools such as the Bayley Scales of Infant and Toddler Development may be influenced by participant cooperation, may not strongly correlate with later outcomes, and require adaptation for the local language and culture. Brain function and anatomical changes occur rapidly in early life, highlighting the potential of advanced, objective measures of brain activity and neural processing, such as electroencephalography (EEG), to detect early abnormalities in brain development and deliver interventions during early, sensitive periods of infant neurodevelopment.

In recent decades, studies exploring the possible neural mechanisms underlying associations between adverse life experiences and long-term child outcomes have increased exponentially with the introduction of advanced neuroimaging technologies, such as magnetic resonance imaging (MRI), functional near-infrared spectroscopy (fNIRS), and EEG (Yeung et al., 2017; Blaisdell et al., 2022; Norton et al., 2021). MRI is widely employed in developmental research, offering high spatial resolution and detailed anatomical images of the brain. However, its implementation is constrained by the need for MRI machines and infrastructure that is often unavailable in LMIC, although access is increasing with new initiatives, such as the Kings College London UNITY collaborative (Abate et al., 2024). Meanwhile, fNIRS is a valuable tool to measure brain blood oxygenation and regional activation, however can only measure surface/cortical brain activity which – not reflect deeper brain structures – and has lower spatial resolution relative to MRI.

EEG is a non-invasive neuroimaging technique designed to capture the brain’s regional electrical activity and neural connectivity using small sensors placed on the head. One of the most common EEG metrics used in developmental literature is the EEG spectral power. EEG spectral power refers to the magnitude of electrical brain activity within specific frequency bands and has been shown to be a potential marker of an array of later neurocognitive abilities (Bhavnani et al., 2022; Leal Neto et al., 2021). For example, higher resting-state alpha band power has been shown to be a sensitive predictor of language (Huberty et al., 2023) and cognitive outcomes (Bhavnani et al., 2021); and higher theta power in infancy can be indicative of reduced language and cognitive development (Tan et al., 2024). Power can be expressed as either absolute (energy within a chosen frequency band) or relative (energy within a chosen band divided by the total energy from all bands). The direct measure of brain activity has also been leveraged in another commonly used metric — the event related potential (ERP). ERPs refer to the EEG component that reflects brain responses to an event or stimuli. One ERP paradigm is the visual evoked potential (VEP), which unlike other ERP paradigms (e.g., P300), does not rely on language proficiency or task adaptation for different cultural settings. The VEP can serve as a proxy measure of the functional integrity of the visual cortex via examination of changes in brain electrical activity in response to a visual stimulus, and may also generally reflect synaptic efficiency. Visual cortex function is critical for early brain development, as it provides the primary sensory input for learning and interaction with the environment (Rose et al., 2003). Common outcome metrics of VEP are the magnitude and latency of the first positive peak (P1) component, which have been shown to positively correlate with longitudinal child outcomes amongst typically developing and clinical child populations, including those in LMIC (Bhavnani et al., 2022; Jensen et al., 2019; Katus et al., 2020). The VEP is non-dependent on verbal and motor response, allowing objective measurements among infants as early as two weeks postnatal (Shepherd et al., 1999). Whereas resting EEG power requires minimal setup, less technical expertise, and is more tolerant of movement artifacts—making it highly scalable in LMIC—VEP offers greater specificity in identifying brain responses to direct stimuli, reflecting cognitive processing. Together, these measures provide complementary insight into neural development, both offering a scalable and specific approach to examine early brain function of children in low-resource settings.

EEG offers several advantages compared to other neuroimaging modalities. It has significantly better temporal resolution due to its ability to sample brain activity every few milliseconds. Further, EEG protocols are resistant to motion artifacts and allow for more flexible involvement of caregivers or family members, which may enhance child tolerance. Evidence suggests that EEG can be a highly sensitive tool for examining the influence of adverse life experiences as early as six to nine months of age (Brito et al., 2016; Tomalski et al., 2013). Troller-Renfree et al. (2022) showed that infants of low-income mothers who received a high-cash incentive exhibited higher relative power in the 20-30 Hz band relative to infants in the low-cash incentive group. This demonstrates both the impact of environmental stressors on infant brain activity and the utility of EEG in detecting interventional effects. Finally, because the same EEG procedure can be used from birth across the entire lifespan, it can be incorporated into longitudinal studies without the need to change measures or instruments, making it a promising option for examining child neurodevelopment on a longitudinal scale.

Emerging evidence suggests that certain EEG metrics may be attributed to contextual factors, including culture and socioeconomic status, emphasizing the need for diversifying study populations to better represent research populations (Parameshwaran et al., 2021). However, majority of developmental EEG research has been conducted in high-income settings using lab-based EEG systems. These systems characterized by high costs and the need to record in highly controlled environments using specialized infrastructure, such as shielded chambers. The advent of mobile EEG technology has made it possible to implement such tools in a wider range of settings and populations (Troller-Renfree et al., 2021), including those in LMIC as observed in Bangladesh (Jensen et al., 2019), the Gambia (Katus et al., 2019), India (Bhavnani et al., 2022), and Malawi (Leal Neto et al., 2021).

This paper will summarize our learning experiences in implementing EEG and VEP as part of the Impact of Maternal Antenatal Nutrition and Infection Treatment Interventions Longitudinal Infant Development and Growth (“LIDG,” meaning child in Amharic) study in rural Amhara, Ethiopia (Workneh et al., 2024). Our discussion will encompass challenges and corresponding practical and methodological strategies undertaken with respect to the (1) equipment, (2) environment, (3) culture, (4) administration, and (5) data processing and analytic factors specific to our study.

### LIDG study overview

The LIDG study is a longitudinal infant follow-up examining the neurodevelopmental outcomes of children aged 12, 18, and 24 months, whose mothers participated in an antenatal nutrition and infection management intervention in rural Ethiopia (Workneh et al., 2024). Children were assessed using a comprehensive battery of neurodevelopmental measures, including EEG collected at the 24-month visit.

## Materials, equipment, and methods

The EEG assessment was conducted by two study nurses at local health centers using the Enobio-32 (Neuroelectrics, Barcelona, Spain), a 32-channel mobile EEG system. The Enobio-32 was selected for its portability, relatively lower cost, and previous validation in a large-scale, randomized controlled trial investigating the effects of poverty reduction on infant brain development (Troller-Renfree et al., 2022). We used attractive, child-friendly screen-savers to collect resting-state EEG and a pattern-reversal checkerboard paradigm to collect VEP (Odom et al., 2016). These stimuli have been used in previous studies of infant EEG in LMIC (Jensen et al., 2019).

### Equipment and software

Our decision on the hardware and software components of the EEG system was informed by a review of the literature for studies in comparable contexts and consideration of factors related to feasibility for administration, maintenance requirements, and suitability for our target population.

#### Electrodes

**Figure 1:**
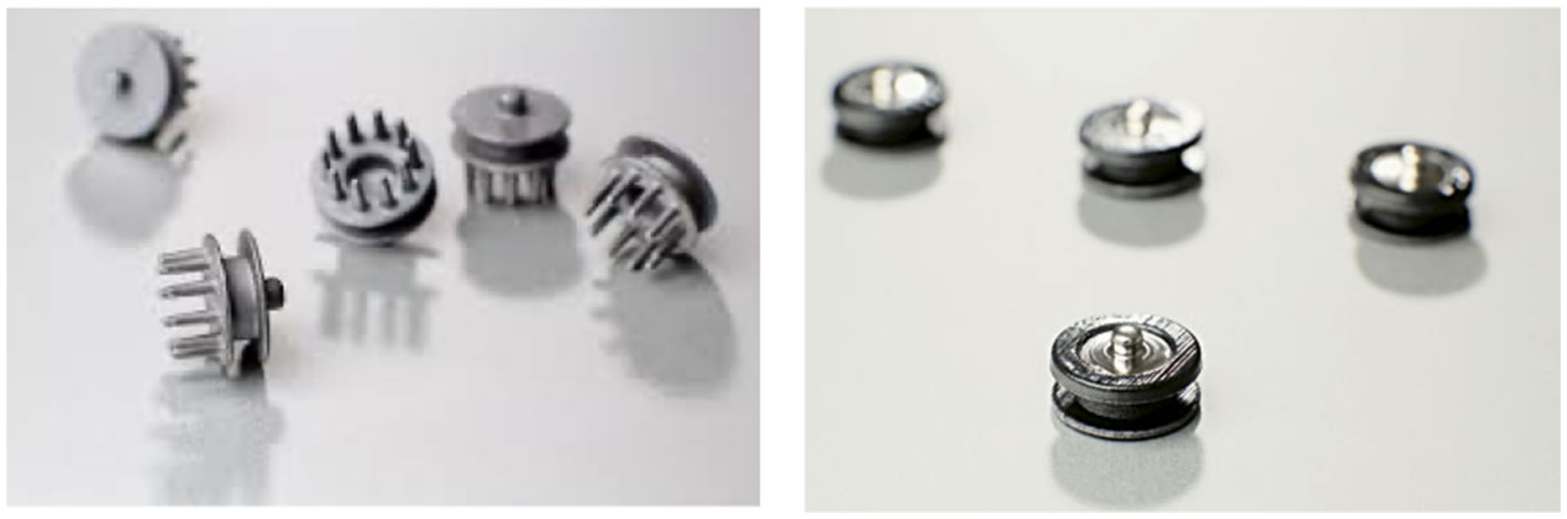
Enobio-32 Drytrodes (left) and Foretrodes (right) (Neuroelectrics, Barcelona, Spain). Source: https://www.neuroelectrics.com/solutions/enobio/32.

Electrical brain activity is measured via electrodes – thin metal disks connected to wires – that are placed on the scalp surface to detect and record changes in electrical signals. Electrodes are broadly categorized as wet or dry and are mainly differentiated by their conductive medium and application methods. Conventionally, wet electrodes rely on a saline solution or conductive gel, and are considered the gold standard in research and clinical applications of EEG. Dry electrodes, in contrast, do not require a conductive medium. Dry electrodes were available in two forms in the Enobio-32 system, including a 10-point contact, comb-shaped silver chloride (Ag/AgCl)-coated electrode (Drytrode), and a flat metal disk electrode designed for use on bare skin (Foretrodes) (Figure 1). For the LIDG study, we elected to use the dry electrodes for several reasons. Firstly, relative to the gel-based electrodes, the dry electrodes required substantially less preparation and cleaning time, which is important considering the limited supply of running water at the study sites. Further, the comb-like appearance of the Drytrodes allowed easier penetration of denser and textured hair types compared to the gel-based electrodes (Adams et al., 2024). Despite previous reports of worse data quality with dry electrode use (Troller-Renfree et al., 2021), we found that manual adjustment of electrodes was sufficient to correct high impedance values and yield acceptable signal quality. This is consistent with a multi-country report demonstrating comparable quality between dry and gel-based electrodes, citing greater variance in electrode performance due to operator experience and training (Ng et al., 2022). Further, the use of dry electrodes reduced the logistical complexity involved with importation of liquid or gel material across international customs.

However, a primary drawback of using dry electrodes is the stringent maintenance procedures required to maintain their quality and conductivity. The Ag/Cl component of the Drytrodes is highly susceptible to corrosion and degradation when exposed to excessive light, moisture, and other metallic material. To extend the electrode lifespan, we established standardized maintenance procedures, including storage in a plastic container separate from other electronic or metallic materials in a dark space, and electrodes were sterilized using 70% ethanol spray due to its quick-drying properties. Further, study staff conducted regular checks for device damage to ensure timely repair.

#### Amplifier connection

Electrodes could be connected to the amplifier via three options: Wi-Fi, Bluetooth, and a physical cord. Consistent with previous reports (Troller-Renfree et al., 2022), pre-study tests revealed unstable signal capture and intermittent connection loss when using Wi-Fi or Bluetooth. For VEP, electrical signals respond in a time-locked manner to the visual stimulus, linking specific brain activity to the event with considerable temporal resolution. To prevent any signal delays or interruptions that could compromise accuracy, we used the physical cord as the primary connection method. However, the physical cord connection also presented several challenges, including greater vulnerability to damage from physical stress due to repeated use, especially at critical connection points between electrodes, the amplifier, and the computer. In our study, multiple device components required replacement and repair, such as a damaged cord and amplifier port. The repair process involved complex steps, including the return shipment of the device from Ethiopia to the manufacturer in Spain, which resulted in a loss of approximately four weeks of data collection time. In consideration of these challenges, study staff were trained to identify damages and associated effects on signal quality, and any concerns were communicated to field supervisors.

#### Cap

**Figure 2:**
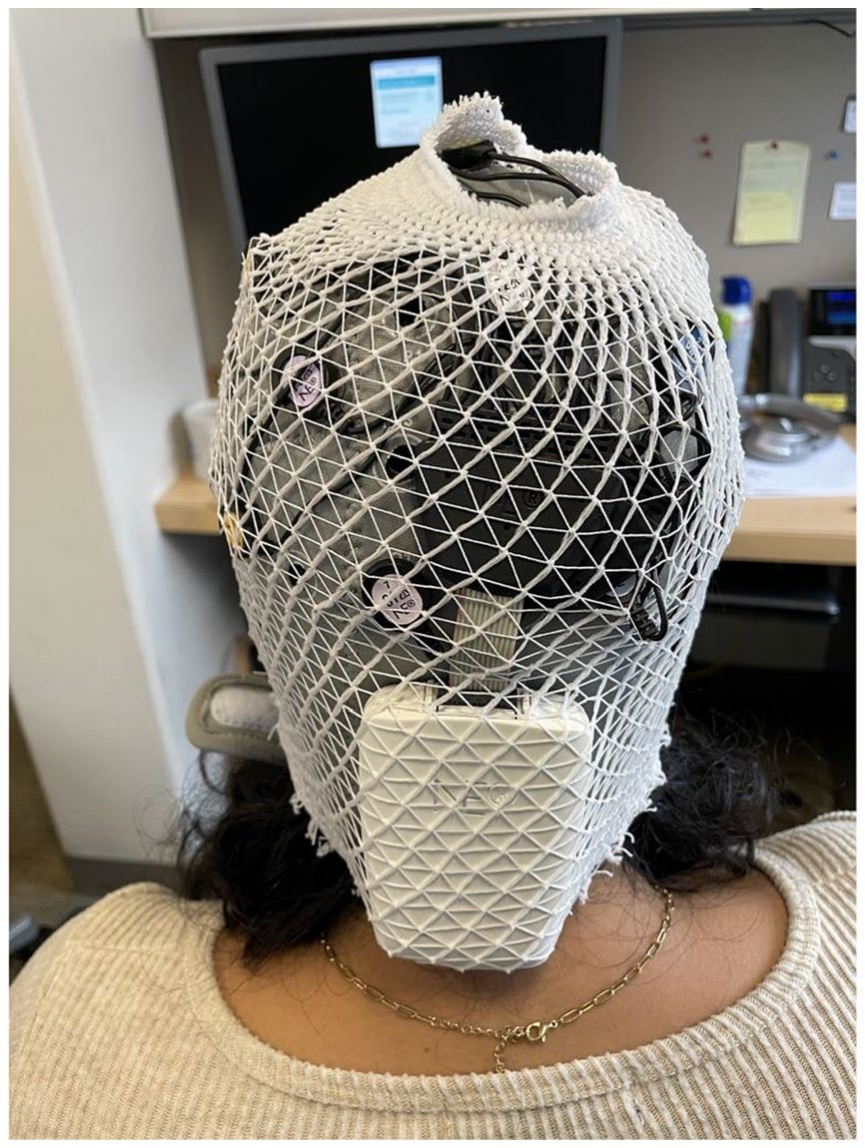
Use of tubular net bandage to provide tighter head cap fit.

The Enobio-32 was equipped with a wearable component consisting of a neoprene head cap with a 39-hole electrode positioning grid designed based on a subset of the 10–20 international system (American Clinical Neurophysiology Society, 2006). Varying cap sizes accommodated cranial perimeters ranging between 42-60 cm. Before recording, we measured each child’s head circumference to determine the appropriate cap size. Among several participants, we observed poor fit on the vertex and posterior regions of the head, which alluded to possible design limitations for particular head shapes. Since most EEG is conducted in Western settings, we speculate that the cap design may be partial to Eurocentric cranial morphology. There is evidence for distinct head shapes between Asian and Caucasian individuals (Ball et al., 2010), which informed the design of A- and C-cut EEG caps (Brain Products GmbH, 2025). However, possible head shape variations amongst individuals of African descent have not been explored. To address these limitations, tubular net surgical bandage (e.g., *Surgilast*) could be placed over the EEG cap to provide a tighter fit and facilitate better scalp contact, as necessary (Figure 2).

#### Computer

The EEG/VEP stimuli were presented using E-Prime 3.0 (Psychology Software Tools, Pittsburgh, USA), a stimulus presentation software, and the recording and visualization of EEG data was conducted using the Neuroelectrics Instrument Controller (NIC2) proprietary software from Neuroelectrics. We adopted a dual-screen configuration, using an external monitor screen for stimulus presentation and a laptop for experiment control and real-time data monitoring. A major lesson from our experimentation was that the computer hardware needed to have adequate computational processing power to support the computational demands of running multiple software, as well as the impacts of delays on the timing accuracy of the presented visual stimuli.

During the initial phases of the study, we used laptops that were compliant with the minimum requirements of E-Prime and NIC2 (Operating system: Windows 10 or 11; Processor: Intel Core i5 3,000 3GHz or AMD FX 6000 Series 3GHz; Random Access Memory (RAM): 8GB; Video card: DirectX 11 or greater; Hard disk: HDD). However, we faced recurrent frozen screens, desynchronized signals, and abrupt shutdown of the recording application. Despite meeting the minimum hardware requirements for each software (i.e., E-Prime and NIC2), using them in tandem generated an overload of computational and graphical processing demand, resulting in recurrent timing delays and reduced sampling rate. To troubleshoot, we upgraded the laptop to meet three primary specifications. We required a higher number of processing cores and threads, such as that available in the Intel i7-13700HX (16 cores and 24 threads). This allowed for dedicated processing of each piece of software and parallel processes that are involved in E-Prime. Secondly, we prioritized a minimum of 32GB of 5,200 DDR5 RAM to allow for more computational multitasking. Finally, the laptop needed to have a dedicated Graphics Processing Unit (GPU) to allow for more independent processing of the physical presentation of the stimuli, which in turn reduced computing stress on the Central Processing Unit. A second strategy was to match the laptop refresh rate with the maximum refresh rate of the peripheral monitor. Following these strategies, we eliminated the aforementioned issues and were able to seamlessly record EEG data. Specific to the VEP data collection, the selected computer hardware needed to have sufficient processing power to support the computational demands of running multiple software, as well as impacts on timing accuracy.

For task presentation, we used an external monitor to display the resting task and visual stimuli. Although the use of external monitors facilitated a more controlled experimental setting, several disadvantages were noted. Firstly, the monitors lacked an internal power source, thus requiring it to be plugged into a wall outlet if no power supply unit was available. This meant that we could not use it in the event of a power outage. The second disadvantage was that the power running through the power cable was detectable by the amplifier, thus creating line noise.

#### VEP paradigm

Several stimulation paradigms exist for examining VEP in developmental research. Among commonly used paradigms are flash VEPs, which present flashing, patterned or non-patterned stimuli or pattern-reversal VEPs, which typically involve a reversing black-and-white checkerboard. In our study, we used the pattern-reversal visual stimuli. Pattern-reversal visual stimuli produce less variability in responses at both the individual and group level compared with flash VEP. The checkerboard pattern was chosen because of its simplicity and established use within developmental EEG research, supporting its reproducibility. The duration of stimuli was 500 ms or 2/s, consistent with prior VEP research in developmental populations (Varcin et al., 2016; Saby et al., 2023; Webb et al., 2023). Ideally, the system recording EEG should be hardwired to the device presenting the stimulus to ensure precise timing and transmission of stimulus onset markers. These event markers are required to estimate synchrony between the stimulus presentation and the brain response and are crucial for data processing.

During our pilot testing, we explored two software options for stimulus presentation: E-Prime and Presentation (Neurobehavioral Systems, Inc., Berkeley, USA). When comparing the two, we considered the connection protocol between the stimulus presentation software and the hardware of the Enobio-32 amplifier, as well as timing precision (e.g., drift and variations in the timing of stimulus markers). Both the Presentation and E-Prime software uses a Lab Streaming Layer (LSL) connection to stream and synchronize EEG data. We encountered several issues for both E-Prime and Presentation. Whereas LSL was integrated within the Presentation software, E-Prime required a third-party software downloaded from GitHub, called LabRecorder, to connect with LSL. Initially, when using E-Prime, we faced difficulties in establishing a stable connection between LabRecorder and the LSL. Attempts to connect often resulted in errors, causing E-Prime and the computer to freeze, requiring a full system reboot. This issue was later resolved following a new software update for the NIC2, which allowed for a direct connection to LSL without LabRecorder. Although we did not observe any LSL connection issues when using Presentation, we identified a consistent timing drift, which affected the time window for data processing due to the mis-marking of the time during which stimulus markers were dropped. Based on our findings, we elected to use E-Prime as our primary stimuli presentation software. Noteworthy is the increased training burden as non-specialist study nurses were required to gain competency in two separate software.

Our comparison of E-Prime and Presentation software highlighted the challenges and solutions in achieving reliable connectivity and timing. While E-Prime required additional setup initially, updates ultimately provided a more stable and accurate system, whereas timing drift in Presentation posed limitations. These findings underscore the importance of selecting and optimizing tools for precise VEP collection.

### Environment

#### Assessment set-up

**Figure 3:**
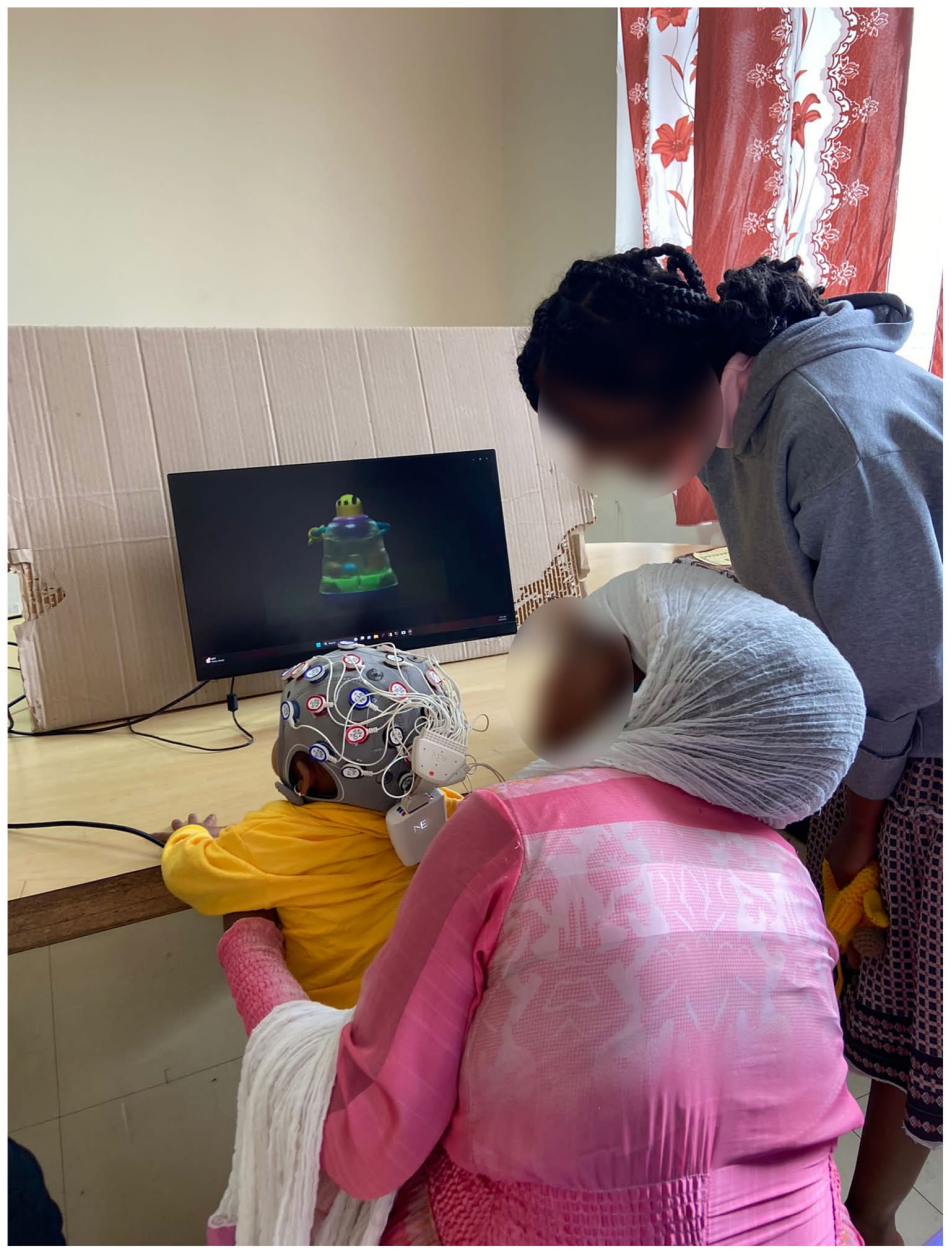
Modified EEG set-up at a local health center from the child’s viewpoint.

EEG recordings were conducted in the LIDG study health centers, which also serve as primary healthcare facilities for the local community. Due to the large volume of patients, we conducted recordings in a designated private room away from foot traffic to minimize noise and disruption. No other assessments were concurrently conducted during the EEG recording. In contrast to higher-resource settings where EEGs are typically conducted in dual-room set-ups, with separation of assessment and experiment control environments, health facilities in LMIC present a challenge of space constraints, limiting the ability to conduct EEG in a separate, controlled setting. For the LIDG study, we simulated a dual-room setup by using a divider (e.g., cardboard) to separate the assessment (child-facing) environment and the experimental control (assessor-facing) environment (Figure 3). It was critical that assessors were not within the child’s view to minimize distractions. In addition, we also collected information regarding sources of possible background noise (e.g., excessive child movement, use of power generators) to aid data cleaning procedures.

#### Line noise

In Ethiopia, electricity is inaccessible to more than half (~60%) of the population, and stark urban–rural disparities in electricity access are reported (95% vs. 27% in urban and rural regions, respectively) (MoWIE, 2019). Additionally, a majority of households in Ethiopia experience frequent power shutdowns, leading to reliance on off-grid solutions, such as generators, for electricity. Recording EEG while relying on generator power could lead to challenges in data processing. Line noise is a major source of artifact in EEG signals, thus removing it from EEG is one of the key steps in EEG preprocessing. Traditionally, with a known line noise frequency (e.g., 50 Hz in Ethiopia and 60 Hz in the US), a notch filter could be applied to suppress the power of line noise in EEG. More recently, CleanLine (Bokil and Mitra, 2007) – a line noise removal method that adaptively estimates and removes sinusoidal noise from EEG signals using multi-tapering – has become more popular. This method is particularly useful when the exact frequency of line noise is unknown. Users can specify a narrow band – usually a few hertz around the presumed line noise frequency – for the algorithm to search for line noise and adaptively remove it.

**Figure 4:**
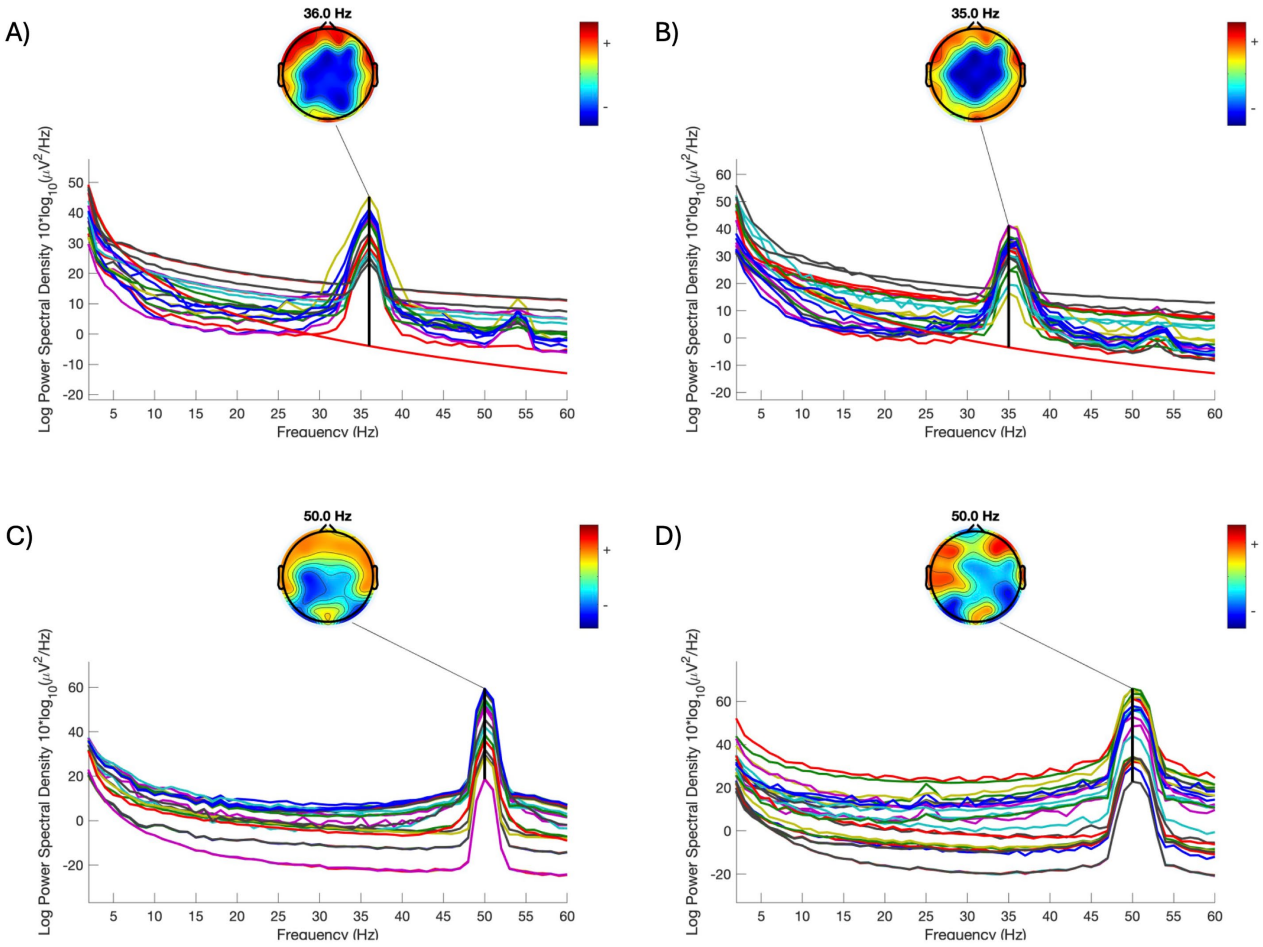
Comparison of power spectral density of individual EEG signals acquired on different power sources. **(A,B)** Two individual examples when EEG recording was powered by a generator. **(C,D)** Two individual examples when EEG recording was powered by grid. **(A–C)** were collected on the same day; **(D)** was collected one day after the other recordings. All data was collected at one of the study health centers. Each line in each subfigure represents a signal in an electrode. Topomaps are plotted for the frequency of line noise in each subfigure.

However, when an EEG recording is powered by a generator instead of a grid, the dominant line noise can vary substantially from its desired frequency. Figure 4 illustrates two example recordings (A and B) where EEG was collected while on the generator. The dominant line noise frequencies are 42 Hz and 41 Hz, respectively – both are far from 50 Hz (C and D) and are beyond the narrow search band (e.g., 48–52 Hz) commonly used in CleanLine. This requires processing parameters to be customized for each recording. Additionally, line noise in a low-40 Hz range overlaps with the “gamma band” activities – a higher-oscillation frequency band (typically 30–50 Hz in EEG). Introducing a notch or low-pass filter to remove line noise within this frequency range will inevitably bias the calculation of gamma-band power, and therefore the examination of gamma and the filter settings requires extra caution.

### Cultural considerations

#### Community perception and acceptability

Prior to study initiation, we conducted formative research through in-depth interviews among community members (*n* = 16) (e.g., mothers, fathers, community leaders) and healthcare providers (*n* = 24) from four of the LIDG study health centers. We explored the perceptions and acceptability of neuroimaging techniques. Detailed procedures and results are reported elsewhere (Workneh et al., 2025).

#### Hair type

Historically, traditional EEG methods using the application of conductive gel have led to the exclusion of participants of African descent in EEG research. Studies suggest that due to the largely textured or dense hair types among this population, there is greater difficulty in ensuring gel penetration to the scalp (Choy et al., 2022) and the use of more gel may lead to possible electrode bridging issues (Webb et al., 2022). The use of Drytrodes (Figure 1) in our study provided substantial advantages to improve electrode contact with the scalp and collection of high quality EEG signal. We required that 26 channels had good signal as indicated by a quality index indicator on the NIC2 software prior to initiation of the EEG recording. In the study site, children aged 24 months commonly have short and sparse hair, and common hairstyles include cornrows. In addition to using dry electrodes, we asked mothers to style their child’s hair in low braids or hairstyles that do not result in excessive hair thickness at the back of the head to facilitate optimal scalp contact, which is particularly crucial for VEP acquisition. Additionally, mothers were also informed to wash their child’s hair the day prior to the visit and to avoid applying any hair oil before the visit to ensure optimal conductivity.

### Administration strategies

#### Training

In the LIDG study, EEG and VEP were administered by non-specialist users who were composed of four Ethiopian study nurses who had no previous experience in neurocognitive or neuroimaging assessments. The core training curriculum involved a two-week, intensive training consisting both didactic and practical sessions. Didactic training was conducted in a classroom setting, and involved a two-day seminar to introduce concepts of EEG for developmental research and general EEG methodology, followed by a four-day workshop focused on technical steps to record EEG/VEP using Enobio-32, data quality checks, child behavioral management techniques, and device maintenance procedures. During the workshop, study nurses conducted mock EEG/VEP recordings with one another. In the second week, we conducted practical training sessions at local health centers in Addis Ababa among children from the local community who were of similar age to the LIDG participants. Practical training sessions were conducted during times that coincided with routine nutrition and vaccine appointments at the health center. Each study nurse was required to complete a minimum of four EEG assessments and two training certifications. Practical training sessions were supervised by research staff who were experienced in EEG implementation. To be eligible to administer EEG for the LIDG study, study nurses were required to satisfy all items on the training certification checklist, which encompasses correct hardware preparation (e.g., device charge at >70%), capping procedure and positioning (e.g., cap is not too low on the forehead), minimum signal quality threshold met (e.g., >80% of electrodes showed good quality indicator), and correct device storage and maintenance (e.g., cap is sanitized). We also conducted a refresher training session and regular consultations to improve data quality between EEG experts and local field teams. One of the key challenges we faced was the limited ability to conduct routine data quality monitoring. Due to ongoing civil conflict and limited internet access in the rural Amhara region, data upload to the shared server was consistently delayed. Therefore, supervision was limited and any identified administration errors or data quality issues could not be addressed promptly. To address these challenges, we developed illustrative job aids to help ensure the correct administration of EEG/VEP, along with common troubleshooting methods.

#### Child behavior management

Collecting good quality EEG data among young children is well known to present several practical challenges. The first challenge is the reduced behavioral repertoire exhibited by young children, particularly among those who are pre-verbal. At 24 months, children in the LIDG study were active and frequently attempted to reach for EEG cables, which introduced significant movement artifacts. Another challenge is children’s limited attentional capacity. This is especially of concern for the VEP paradigm, which requires children to attend to the stimulus on the screen for accurate data capture. Several strategies were implemented to help minimize these challenges. Firstly, study nurses were trained on various child behavior management strategies, including modulating their behavior and tone of voice to suit each child. Study nurses were encouraged to welcome children using child-friendly language to help them acclimatize to the assessment environment. Strategies included communicating with children by asking simple questions (e.g., “What is your name?,” “Would you like to look at a few toys?”) and allowing children to explore the assessment room before initiating the study visit. To reduce the pulling of EEG cables, children were given multiple toys to hold (e.g., one in each hand) or toys designed to be held with both hands. Within the local community, we observed that children were disinterested in battery-operated electronic toys (e.g., musical steering wheel) likely due to lack of prior exposure to such toys. Similarly, attractive short animated videos on the laptop were often ineffective in distracting children during the capping process. Instead, we found that balls, small cars, and stuffed animals, which are more widely available in these settings, were more effective in engaging children.

In rural Amhara, children preponderantly displayed shy, reserved behaviors, and frequently looked to their caregivers for behavioral cues. During EEG recording, mothers were encouraged to utilize silent gestures (e.g., pointing) to encourage children to engage in the EEG tasks. If children were distracted during the EEG recording, study nurses would use various colorful and sound-making toys (e.g., stuffed animal, xylophone) to draw the child’s attention to the screen. In tandem, manual keyboard markers were programmed and entered to track the child’s gaze behavior (attending vs. not attending to the task on the screen) for later data cleaning. Specifically, the study nurse on the child’s side used hand gestures to signal attending vs. non-attending behavior to the study nurse observing the EEG recording for manual marking.

#### Cap application

During the formative work, safety emerged as a primary concern among community members. Due to the use of dry electrodes, children may feel slight discomfort from the pressure of the cap, which may lead to worries about EEG safety among caregivers. One of the strategies implemented to minimize discomfort was the cap application method. While the child was seated on their caregiver’s lap, the two study nurses were positioned in front of and behind the child, respectively. Once the child was calm, the study nurses simultaneously stretched the front and back sides of the cap and lowered the cap onto the child’s head. This strategy helped minimize the downward dragging of the comb-shaped electrodes along the scalp, which may cause slight discomfort to infants but is not harmful.

The Enobio-32 system was equipped with ear clips for the application of the ground/reference Common Mode Sense (CMS) and Driven Right Leg (CMS/DRL) electrodes. We chose to apply the CMS and DRL electrodes using adhesive sticker electrodes (Kendall ECG electrodes H124SG, Covidien LLC, Mansfield, USA) onto the child’s earlobe and found that children were less prone to distress and pulling off the electrodes compared to when ear clips were used.

### EEG pre-processing

Resting EEG data were pre-processed using miniMADE (Troller-Renfree et al., 2021), a standardized EEG preprocessing pipeline that was modified from the original MADE pipeline for low-density and mobile EEG systems (Debnath et al., 2020). Advanced algorithms like Fully Automated Statistical Thresholding for EEG Artifact Rejection (FASTER) (Nolan et al., 2010) and Independent Component Analysis (ICA) (Delorme and Makeig, 2004) as well as standard processing steps including interpolation and common average reference were excluded in miniMADE as they typically require high-density recordings, more standardized environments, and less noise for optimal performance. Preprocessing parameters recommended in Troller-Renfree et al. (2021) were used. More specifically, EEG signals were first downsampled to 250 Hz. Bandpass filtering with cutoff frequencies [0.3, 30] Hz was applied. Continuous EEGs were then segmented into overlapping 1-s epochs and re-referenced to the average of T7 and T8 electrodes. Epochs were examined for eye blinks and extreme values. If both prefrontal channels (FP1 and FP2) contain values exceeding [−250,250] μV during an epoch, suggesting an eye blink during this window, this epoch was removed; otherwise, all other channels were assessed by the same threshold – channels with values beyond [−250,250] μV were filled with NaNs. Notably, this thresholding allows unequal numbers of usable channels across epochs, which helps maximize the amount of data retained. After amplitude-based exclusions of artifactual data, if an epoch contained more than 20% of missing channels (i.e., whose signals were replaced with NaNs), the entire epoch was rejected from further analysis. After preprocessing, we conducted a power spectra analysis using MNE-python (Gramfort et al., 2014). Two regions of interest (ROIs) were defined: a frontal ROI including electrodes Fz, FC1, FC2, AF3, AF4, F3, and F4, and a posterior ROI including electrodes Pz, PO3, PO4, O1, Oz, O2, P3, and P4.

VEP data were preprocessed using the Harvard Automated Pre-processing Pipeline for EEG (HAPPE, version 3.3) (Gabard-Durnam et al., 2018). Specifically, line noise (50 Hz) was first removed using CleanLine (Bokil and Mitra, 2007) via a multi-taper regression approach. Signals were then resampled to 250 Hz and lowpass filtered (100 Hz). Bad channels, including those with a flat line, residual line noise, and other excessive noise evaluated by a joint probability method, were removed and interpolated. This was followed by a wavelet-thresholding artifact removal pipeline that decomposes signals into frequency components and identifies artifacts based on the distributions of these components. A bandpass filter (1–30 Hz) was then applied to remove slow drifts and high-frequency noise. Sections of VEP recordings with non-looking behavior were previously marked during data collection by the experimenter; these sections were removed from further processing. Continuous EEG data were then segmented into epochs 100 ms before and 500 ms after each stimulus onset; baseline correction was applied using signals in the pre-stimulus window. EEG epochs were re-referenced to the average of electrodes T7 and T8. Epochs with extreme values (outside 250 μV), indicating excessive residual noise, were finally removed.

## Results

### Knowledge, perception, and acceptability of EEG

Broadly, participants had limited knowledge of EEG, with no participants reporting any previous experience with EEG assessments. Most participants perceived EEG procedures to be easy, safe, and beneficial to children. Several parents reported that EEG is a helpful tool for monitoring children’s development, and one clinician reported that, relative to MRI, the use of EEG may be less anxiety-inducing for parents [“*…the infant does not need to be put to sleep; the procedure can be done while the child is awake and moving. The child will also be on the lap of the mother and not put in the machine. Therefore, it will lessen the mothers’ anxiety*”]. However, several concerns were raised regarding the safety of EEG, including risks of electricity shock, radiation, and possible injuries from EEG electrodes and cap wear [*“I was worried about the dots, whether they would cause injury or not?*”]. A key facilitator of EEG participation is caregivers’ interest in learning about their child’s development. Several participants reported that understanding their child’s development is a key motivator for willingness to participate. In contrast, a barrier to participation included women expressing the need to obtain permission from their husbands prior to consenting for their child to undergo EEG.

Overall, our study showed that EEG was acceptable among families, demonstrated by a high rate of consent of 257 out of 264 (97.3%) eligible participants. The findings of the formative work emphasize the importance of creating awareness and engaging fathers, community representatives such as religious leaders, and healthcare providers to create community buy-in. Utilizing the findings of the formative work, we refined educational materials to address concerns and enhance understanding of EEG safety and benefits. These educational videos were later shown to mothers as part of the consenting procedures and study sensitization.

### Resting EEG and VEP

**Figure 5:**
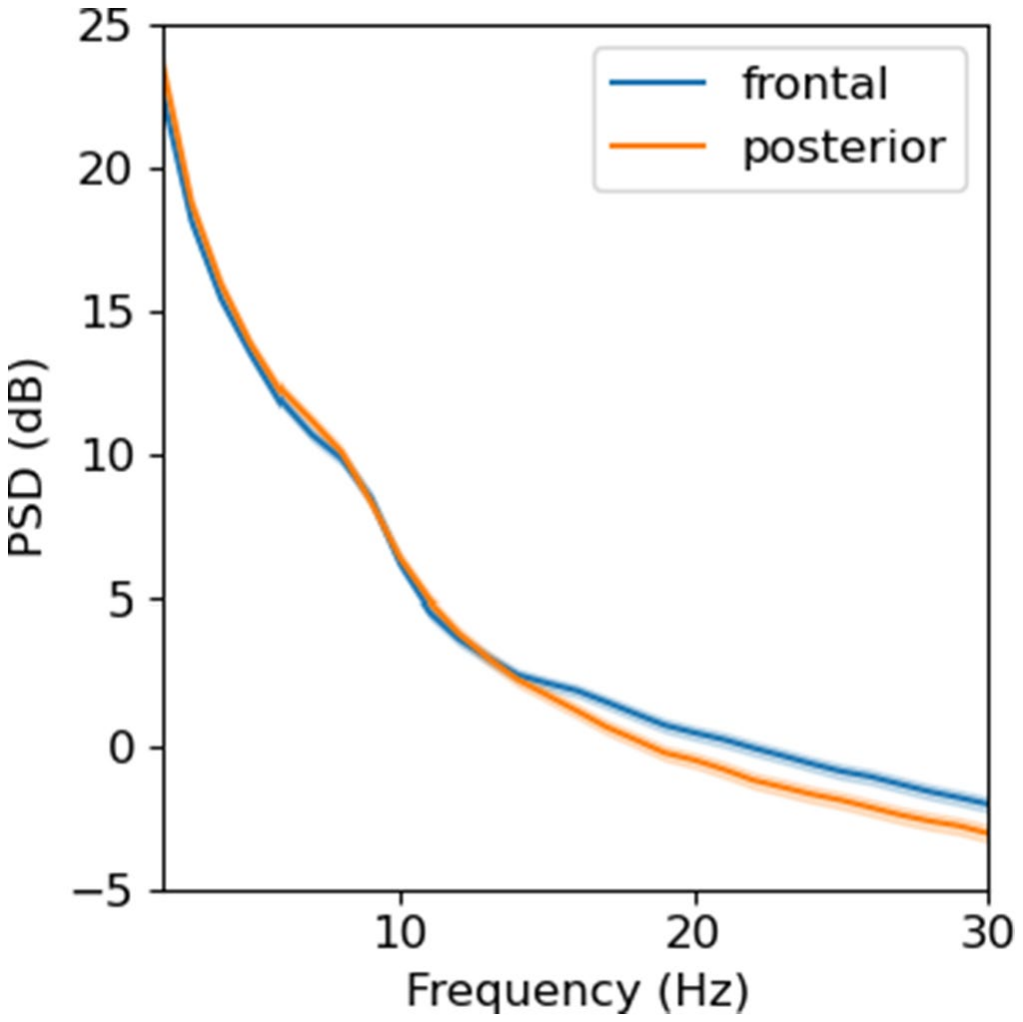
Power spectral density from resting-state, eyes-opened EEG averaged across electrodes in two regions of interest (*N* = 173).

In total, we collected 187 resting EEG and 177 VEP recordings. The average EEG protocol duration was 20 ± 11 minutes, measured from the time a child was capped to the time the cap was removed. For resting EEG, 174 (93%) recordings met data quality thresholds, among which 173 (99%) had a minimum of 30 epochs after artifact rejection. Using Welch’s method (Hamming window, 256-point FFT, no overlapping), we observed a power spectral density (PSD) with a 1/f characteristic and a “bump” between 6–9 Hz in both ROIs (Figure 5). The shape and magnitude of the PSD were similar to what has been previously reported in developmental EEG studies (Rodríguez-Martínez et al., 2020; Roche et al., 2019).

**Figure 6:**
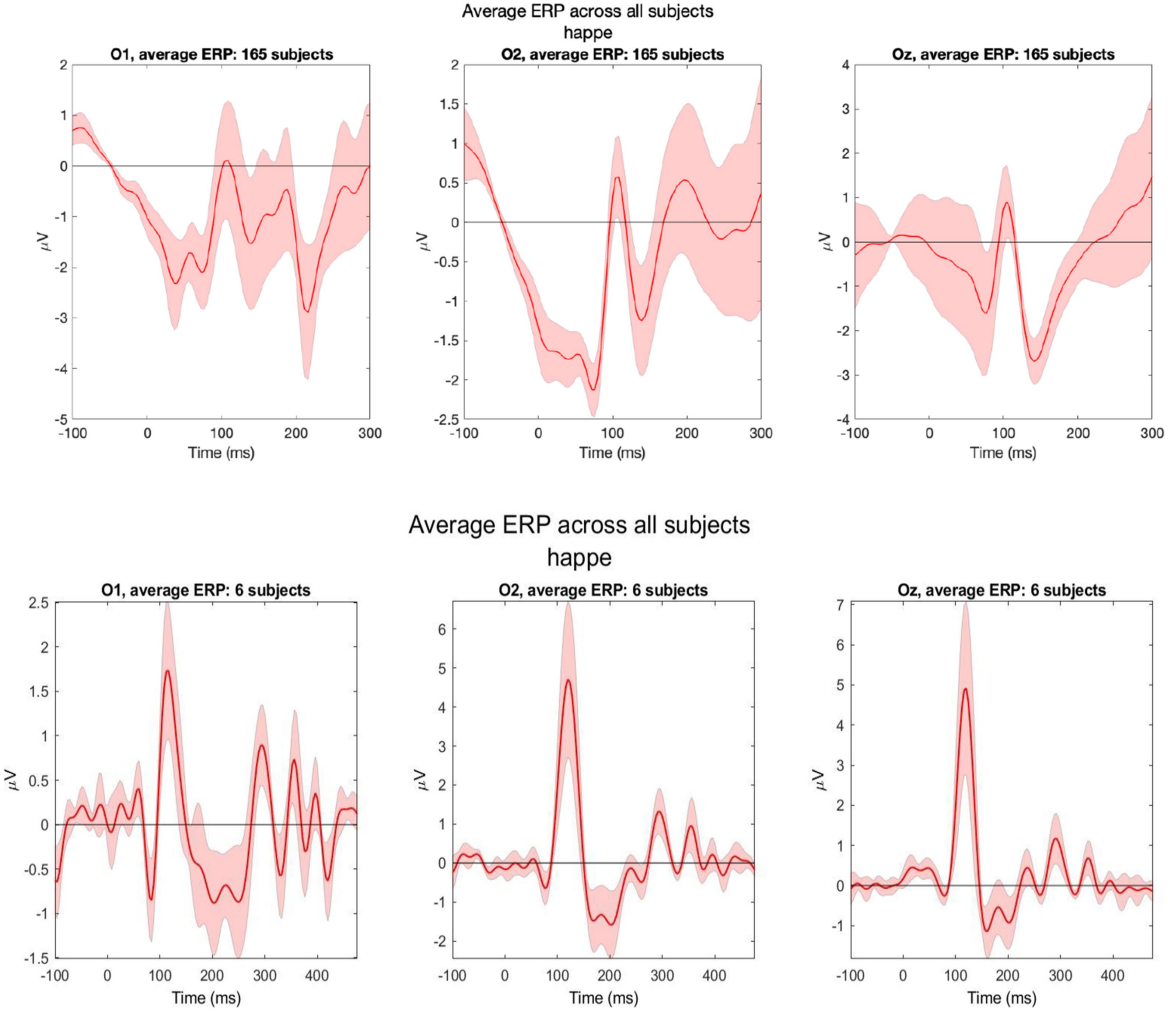
Grand average VEPs from three occipital electrodes (O1, O2, and Oz). Data were collected from 165 LIDG participants by non-specialist study nurses (top) and two adult volunteers (three recordings per volunteer) during pilot testing by EEG experts in Boston (bottom).

For VEP, 165 out of 177 recordings were retained post-processing (93%). We analyzed data from occipital electrodes O1, O2, and Oz. Clear N1 and P1 components of VEP were observed in these channels. The VEP morphology observed within our study cohort collected by trained study nurses was comparable to pilot VEPs conducted on two adult volunteers by EEG experts at the Lab of Cognitive Neurosciences in Boston (Figure 6). After upgrading the computer hardware, we found that 70% (vs. 30% before hardware upgrade) of participants had a visible VEP waveform in at least one occipital electrode.

## Discussion

This study contributes to a growing body of literature describing the use of advanced neuroimaging tools in LMIC, where existing guidance informing best-practice data acquisition methods are sparse. In this paper, we offer insights into implementation of EEG and VEP in rural Ethiopia, discussing key methodological, practical, and ethical lessons from our experiences in implementing EEG/VEP among 24-month-old children in this setting.

### Practical and methodological strategies for EEG implementation

Our findings are aligned with previous EEG research conducted in LMIC, including Bangladesh (Jensen et al., 2019), the Gambia (Katus et al., 2020), Malawi (Leal Neto et al., 2021), and India (Bhavnani et al., 2022), which collectively demonstrate the feasibility of implementing EEG in field settings. Similar to these studies, we adapted our methodological approach to address the unique environmental and logistical challenges of our context.

In the LIDG study, we implemented a number of tailored strategies to optimize both data quality and participant tolerance. These included maintaining spare devices and accessories, extending equipment warranties to mitigate hardware durability issues and using computers with advanced technical specifications (e.g., higher RAM and standalone GPU). We also employed culturally-tailored approaches to enhance comfort and compliance during EEG administration, such as styling children’s hair in low braids, using locally relevant toys to manage child behavior, and engaging local community members to foster trust and improve EEG uptake. Interestingly, children in our study setting were not motivated by computer-based videos or electronic toys, tools that have been shown in previous studies to effectively engage children (Bhavnani et al., 2022). This may reflect the contextual differences in prior exposure to digital media, cultural norms surrounding play, and attitudes towards unfamiliar materials between LMIC settings. These findings underscore the importance of understanding the cultural framework in which assessments are to be implemented.

The tailored approach of our EEG implementation culminated in reduced administration time and high data retention rates. The EEG protocol for the LIDG study was completed within an average duration of 20 minutes. This is comparable with protocols from large-scale, multi-center studies from high-income countries such as the Healthy Brain and Child Development (HBCD) study (Fox et al., 2024). Minimizing data collection duration is essential in developmental populations as it minimizes participant burden and supports sustained cooperation while maximizing acquisition of good-quality data. Additionally, we achieved data retention rates of ~90% for resting EEG and ~70% for VEP post-processing, which is comparable to data collected by specialist users as demonstrated in this study and other mobile EEG research (Katus et al., 2019). One notable limitation is the inability to track the child’s gaze behavior. Lab-based EEG setups often incorporate integrated eye-tracking technology (e.g., such as the Tobii eye-tracking software) or gaze-contingent stimuli that are only presented when the child is viewing the screen to facilitate artifact correction and eliminate the need for manual visual coding for data cleaning. Despite studies showing reduced drop-out rates and greater data capture among young children (Haartsen et al., 2021; Ahtola et al., 2017), participants in our study setting were not amenable to the use of cameras due to privacy concerns.

In an experimental setup with excessive environmental noise, atypical line noise characteristics, and possibly imperfect electrode contact, preprocessing of EEG becomes extremely important for optimizing data quality. The current study employed minimal preprocessing steps (down-sampling, filtering, and re-referencing, plus wavelet thresholding only for the VEP paradigm), mostly due to the limited number of electrodes. Advanced denoising techniques, such as blind source separation (BSS) work more optimally with a higher number of channels to leverage spatial information for isolating artifacts (Lopez et al., 2022). In future studies where high-density EEG becomes available, BSS techniques, like ICA (Delorme and Makeig, 2004), can be considered to separate brain signals from artifacts such as eye movement, muscle activity, and environmental noise. A higher number of electrodes, due to its denser spatial sampling, will also enable the application of interpolation techniques to replace electrodes with excessive noise.

### Ethical considerations and community engagement

EEG remains a relatively novel tool within the field of developmental neuroscience research in LMIC, and building a community-informed approach is critical for ensuring community buy-in and a culturally-tailored study design. Our formative findings revealed that a majority of participants responded positively to EEG, primarily motivated by the desire to improve their child’s health and development. However, several participant responses alluded to possible therapeutic misconceptions, suggesting that participants associated receiving EEG with direct developmental benefits to their children. Addressing these misconceptions requires well-defined guidelines consenting and counseling participants in research, including offering parents opportunities to ask questions, communicating the broader purpose of research, and emphasizing that EEG procedures do not confer any direct clinical benefit. The study also raised an important ethical consideration in regards to identifying potential manifestations of clinical conditions in EEG patterns in real-time, particularly given that data acquisition is conducted by study staff without clinical training. Establishing a protocol for management of such incidental findings that addresses timeliness of intervention, clinical responsibility, and patient referral is crucial, especially in settings with limited access to specialized care.

Importantly, insights from the formative study highlight the potential for future studies to benefit from intersecting principles between health and sociology in the design of study protocols. One example is the crucial role of fathers in the counselling and consent procedures. Several mothers expressed requiring permission from their husbands prior to their child’s participation in EEG research. This finding is similarly observed in Lockwood-Estrin et al. (2022), who reported that mothers were more willing for their children to participate in EEG if their husbands also agreed. Together, these findings broadly reflect the cultural practice and unique familial dynamic commonly observed in more collectivist societies, such as Ethiopia, where decision-making is often conferred to male heads of households. As discussed in Shen et al. (2021), such formative work that prioritize engagement with key community stakeholders and better understanding of local contexts is highly beneficial in strengthening local partnerships and research uptake.

Furthermore, our findings contribute to the growing body of literature demonstrating the feasibility of collecting high-quality data using mobile EEG, thereby supporting its potential for scalability beyond traditional laboratory environments, including in educational settings. This presents a promising future direction considering the emerging evidence linking EEG markers with key cognitive and socioemotional domains in infancy and early childhood, including executive functioning, attention, memory, and visual processing (Bhavnani et al., 2021; Katus et al., 2020). Implementation of EEG in educational environments may also offer the opportunity to collect more ecologically valid data, facilitating the mapping of typical neurodevelopmental trajectories in real-world contexts and examine associations with school performance. Moreover, conducting EEG assessments in more familiar and accessible contexts such as schools may help address transportation challenges identified in formative research, thus reducing participant burden and subsequently improving participation. Our study supports previous work demonstrating that EEG and VEP can be feasibly implemented by non-specialist users in a rural LMIC field site while still achieving high-quality data collection. This is critical for advancing efforts to build capacity in neuroimaging for research, clinical care, and developmental assessment in low-resource settings.

## Data Availability

The raw data supporting the conclusions of this article will be made available by the authors, without undue reservation.
